# 

**Published:** 1892-08-06

**Authors:** 


					WITH EXTRA NURSING SUPPLEMEN
Per Annum, 8s. 6d., post free.
Monthly Farts, 6d.; post free, 8d.
Ditto per Annum, 8s., post free.
306. Vol. xn.] Saturday,
August 6th, 1892.
[Twopence.

				

